# A Comparison of TIMI (Thrombolysis in Myocardial Infarction) and GRACE (Global Registry of Acute Coronary Events) Scores, Myocardial Blush Grade, and Shock Index-Based Indices for Predicting Outcomes in STEMI Patients Undergoing Reperfusion

**DOI:** 10.7759/cureus.84938

**Published:** 2025-05-27

**Authors:** Aravind SR, Yashwanth Lakshmaiah, Krishna Moorthy DGSR, Murali Mohan NT, Devendra Prasad KJ, Rajesh K, Nikhil Reddy Y

**Affiliations:** 1 Emergency Medicine, Sri Devaraj Urs Medical College, Kolar, IND; 2 Cardiology, RLJ Narayana Hrudayalaya Healthcare, Kolar, IND

**Keywords:** grace score, myocardial blush grade, risk stratification, shock index, stemi, timi score

## Abstract

Background and objective

ST-segment elevation myocardial infarction (STEMI) is a leading cause of morbidity and mortality globally. Proper risk stratification is critical to guide its early management and forecast outcomes. Although traditional scoring systems such as Thrombolysis in Myocardial Infarction (TIMI) and Global Registry of Acute Coronary Events (GRACE) are commonly employed, more recent indices such as Shock Index (SI), Modified Shock Index (MSI), and Age Shock Index (Age SI), and Myocardial Blush Grade (MBG) provide potentially more straightforward, bedside-usable means for prognostication. This study aimed to evaluate and compare the predictive validity of SI, Age SI, MSI, and MBG with validated risk scores - TIMI and GRACE - in predicting in-hospital and 30-day outcomes in STEMI patients who are undergoing reperfusion therapy.

Methods

This prospective observational study was conducted from September 2023 to August 2024 at R.L. Jalappa Hospital and RLJ Narayana Hrudayalaya, Kolar. A total of 150 adult STEMI patients treated with thrombolysis or primary percutaneous coronary intervention (PCI) were enrolled. Data were examined using chi-square tests and receiver operating characteristic (ROC) curves. Outcomes were assessed as in-hospital mortality, length of stay in ICU, and 30-day major adverse cardiac events (MACE).

Results

GRACE score evidenced higher predictive capacity for mortality and MACE over TIMI and shock-based indices. Age SI and MBG indicated encouraging correlation with outcomes, especially when employed in combination with conventional scores.

Conclusions

GRACE is a potent risk stratification tool for STEMI. Yet, SI-based metrics and MBG may supplement conventional scores, thereby providing fast, bedside assessment to facilitate early intervention and enhance patient outcomes in these patients.

## Introduction

Acute coronary syndrome (ACS) is one of the leading causes of cardiovascular morbidity and mortality globally, and ST-segment elevation myocardial infarction (STEMI) is a dangerous and life-threatening presentation of the disease [1]. Accurate and timely risk stratification is necessary in the treatment of STEMI patients to guide therapy, maximize healthcare resource use, and optimize clinical benefits [2]. A number of risk-assessment tools, including the Thrombolysis In Myocardial Infarction (TIMI) score and the Global Registry of Acute Coronary Events (GRACE) score, have been widely validated and incorporated into clinical practice for this purpose [3-10]. While their prognostic value has been widely reported, the complexity and need for multiple clinical and laboratory parameters often limit their use for immediate bedside application, especially in the emergency department.

On the other hand, Shock Index (SI), the ratio of heart rate (HR) to systolic blood pressure (SBP), gives an immediate, rapid, and handy bedside estimation of hemodynamic status and prediction of impending clinical worsening [11,12]. Derived scores such as Modified Shock Index (MSI), using mean arterial pressure (MAP) rather than SBP, and Age Shock Index (Age SI), multiplying SI with the patient's age, are potentially excellent predictors of complications in STEMI patients [2,13-15]. These scores take vital signs and demographics into account and are therefore particularly appealing to use for early risk stratification in the acute setting, where rapid decisions are paramount. Another tool becoming more widely utilized for assessment of myocardial perfusion is the Myocardial Blush Grade (MBG), a subjective angiographic measure assessing microvascular flow to the infarcted myocardium in response to reperfusion therapy [16]. Whereas traditional risk scores are geared toward highlighting system-level predictors, MBG feeds back immediately as to whether reperfusion to the myocardial level is being achieved despite resumption of epicardial coronary flow. Hence, it is a helpful complement to monitor and direct post-procedure clinical judgment.

Although each of these alternative scoring systems and indices - TIMI, GRACE, SI-based indices, and MBG - has been individually validated by multiple studies, there is sparse comparative data that evaluate their relative accuracy to predict clinical outcomes in STEMI patients treated with reperfusion therapy. Additionally, most available validation studies are geographically or demographically limited, and there is a need for more generalizable, larger research studies. In light of this, the present study aimed to compare the clinical outcomes of STEMI patients treated with reperfusion therapy based on SI, MSI, and Age SI and their predictive values with the standard TIMI and GRACE scores and MBG. We believe our findings will help determine which index provides the most optimal and useful estimate of short-term (in-hospital) and intermediate-term (30-day) major adverse cardiac events (MACE), thereby facilitating improved risk stratification and treatment planning.

## Materials and methods

Study design and setting

This prospective observational study was conducted at R.L. Jalappa Hospital and RLJ Narayana Hrudayalaya, Kolar. The study was aimed at comparing and evaluating the predictive value of TIMI, GRACE, MBG, and various SI-based scores in STEMI patients receiving reperfusion therapy. The study was conducted over 12 months from September 2023 to August 2024.

Study population

The study population consisted of all patients who presented to the above-mentioned hospitals with STEMI during the study period and subsequently underwent primary percutaneous coronary intervention (PCI) or thrombolysis. The inclusion criteria were patients aged ≥18 years with a confirmed diagnosis of STEMI. Patients with unstable angina, NSTEMI, serious arrhythmias, renal failure, end-stage carcinoma, or STEMI in pregnancy were excluded from the study to ensure a homogenous study population and avoid confounding factors.

Data gathering and calculation of the risk score

On admission, the following clinical, demographic, and hemodynamic parameters were recorded: HR, BP, and age of the patient. These parameters were utilized to compute SI (HR/SBP), MSI (HR/MAP), and Age SI (SI × age). TIMI and GRACE scores were computed for all the patients based on standard clinical criteria [3-10]. MBG was assessed angiographically following reperfusion therapy to evaluate microvascular perfusion in infarcted myocardial tissue.

Calculation of TIMI and GRACE Scores

TIMI STEMI risk score at admission was determined based on nine standard clinical variables: age, history of diabetes mellitus, hypertension or angina, SBP, HR, Killip class upon presentation, body weight, ST-segment elevation or new LBBB on ECG, and time since onset of symptoms. Each of these variables was given a definite point value in line with the original TIMI scoring system, and patients were classified as low risk (0-3), intermediate risk (4-6), or high risk (≥7).

The GRACE risk score was calculated with eight clinical factors: age, HR, SBP, serum creatinine, Killip class, cardiac arrest at presentation, ST-segment deviation on ECG, and increased cardiac biomarkers. Scores were calculated by applying the GRACE prediction model calculator and risk stratification into low (≤108), intermediate (109-140), and high (>140) risk categories for in-hospital and 30-day mortality prediction.

Both GRACE and TIMI scores were computed in a systematic way for all the patients at baseline to establish their prognostic ability for clinical outcomes.

Outcome measurement

Both in-hospital and 30-day MACE, including cardiogenic shock, re-infarction, arrhythmias, and death, were the clinical outcomes of interest. These outcomes were tracked and documented systematically both during hospital stay and during follow-up visits.

Statistical analysis

Statistical analysis was performed with routine statistical software. Categorical variables were analyzed with the chi-square test to determine the correlation between risk indices and clinical outcomes. Continuous variables were reported as mean with standard deviation (SD). Receiver operating characteristic (ROC) curves were plotted to determine the discriminatory power of each scoring system to predict poor outcomes, with the area under the curve (AUC) being a measure of accuracy. Graphical figures, such as bar diagrams and ROC curves, were prepared using Microsoft Excel and Word. A p-value of less than 0.05 was considered statistically significant, in line with standard statistical practice.

## Results

Table 1 presents the baseline characteristics of the study cohort, comprising 150 subjects. A substantial proportion of participants were aged between 50 and 65 years (45.3%). The majority were male (74.7%) and predominantly resided in urban areas (65.3%). A high prevalence of sedentary lifestyle was observed among the participants (69.3%). The most common comorbidities were hypertension (52%) and diabetes mellitus (41.3%). Furthermore, over half of the participants were smokers (56%), and 38.7% reported a family history of coronary artery disease (CAD).

**Table 1:** Baseline characteristics of the participants (N=150) CAD: coronary artery disease

Variable	Category	Values
Age, years, n (%)	<50	42 (28%)
	50-65	68 (45.3%)
	>65	40 (26.7%)
Gender, n (%)	Male	112 (74.7%)
	Female	38 (25.3%)
Residence, n (%)	Urban	98 (65.3%)
	Rural	52 (34.7%)
Lifestyle, n (%)	Sedentary	104 (69.3%)
	Active	46 (30.7%)
Comorbidities, n (%)	Diabetes mellitus	62 (41.3%)
	Hypertension	78 (52%)
	Dyslipidemia	56 (37.3%)
Personal history, n (%)	Smoker	84 (56%)
	Alcoholic	45 (30%)
	Tobacco chewer	38 (25.3%)
Family history of CAD, n (%)	Yes	58 (38.7%)
	No	92 (61.3%)

Table 2 summarizes the clinical and risk assessment profiles of STEMI patients. The anterior wall was the most commonly affected location. The left anterior descending artery (LAD) emerged as the most frequent culprit artery. The mean SI and MSI were 0.72 and 1.12, respectively. Based on the TIMI and GRACE risk scores, the majority of patients were classified as being in the intermediate-risk category.

**Table 2:** Angiographic and risk stratification profile GRACE: Global Registry of Acute Coronary Events; LAD: left anterior descending artery; LCX: left circumflex artery; LMCA: left main coronary artery; MSI: Modified Shock Index; RCA: right coronary artery; SD: standard deviation; SI: Shock Index; STEMI: ST-segment elevation myocardial infarction; TIMI: Thrombolysis in Myocardial Infarction

Variable	Category	Values
STEMI location, n (%)	Anterior	65 (43.3%)
	Inferior	52 (34.7%)
	Lateral	18 (12%)
	Posterior	10 (6.7%)
	Septal	5 (3.3%)
Culprit artery, n (%)	LAD	72 (48%)
	RCA	58 (38.7%)
	LCX	15 (10%)
	LMCA	5 (3.3%)
SI	Mean ± SD	0.72 ± 0.15
MSI	Mean ± SD	1.12 ± 0.28
TIMI risk score, n (%)	Low (0-3)	42 (28%)
	Intermediate (4-6)	78 (52%)
	High (≥7)	30 (20%)
GRACE risk score, n (%)	Low (≤108)	55 (36.7%)
	Intermediate (109-140)	62 (41.3%)
	High (>140)	33 (22%)

Table 3 delineates the primary outcome metrics of the investigation. The mean ICU duration was 3.5 days, with an average of 1.8 days on ventilators. The overall hospital stay averaged 6.2 days. The in-hospital mortality rate documented was 12%, while 30-day MACE occurred in 16% of subjects. Among MACE components, heart failure predominated (6.7%), followed by reinfarction (5.3%), revascularization (3.3%), and stroke (2%).

**Table 3:** Clinical outcomes ICU: intensive care unit; MACE: major adverse cardiac events; SD: standard deviation

Outcome	Values
ICU stay, days, mean ± SD	3.5 ± 2.1
Ventilator days, mean ± SD	1.8 ± 1.2
Total hospital stay, days, mean ± SD	6.2 ± 3.0
Mortality, n (%)	18 (12%)
30-day MACE, n (%)	24 (16%)
MACE components, n (%)	
Reinfarction	8 (5.3%)
Heart failure	10 (6.7%)
Stroke	3 (2%)
Revascularization	5 (3.3%)

Association of risk scores with outcomes

Chi-square analysis showed significant correlations between elevated GRACE scores and mortality (χ² = 18.4, p<0.001) and MACE (χ² = 12.6, p = 0.002). High TIMI scores also showed correlations with mortality (χ² = 9.8, p = 0.007) and MACE (χ² = 7.3, p = 0.026). SI and MBG displayed moderate correlations with mortality (SI: p = 0.032; MBG: p = 0.041) (Table 4).

**Table 4:** Association of risk scores with outcomes GRACE: Global Registry of Acute Coronary Events; MACE: major adverse cardiac events; MBG: Myocardial Blush Grade; SI: Shock Index; TIMI: Thrombolysis in Myocardial Infarction

Risk score/variable	Outcome	Chi-square (χ²)	P-value
GRACE score	Mortality	18.4	<0.001
GRACE score	MACE	12.6	0.002
TIMI score	Mortality	9.8	0.007
TIMI score	MACE	7.3	0.026
SI	Mortality	–	0.032
MBG	Mortality	–	0.041

Predictive performance of risk scores

**Figure 1:**
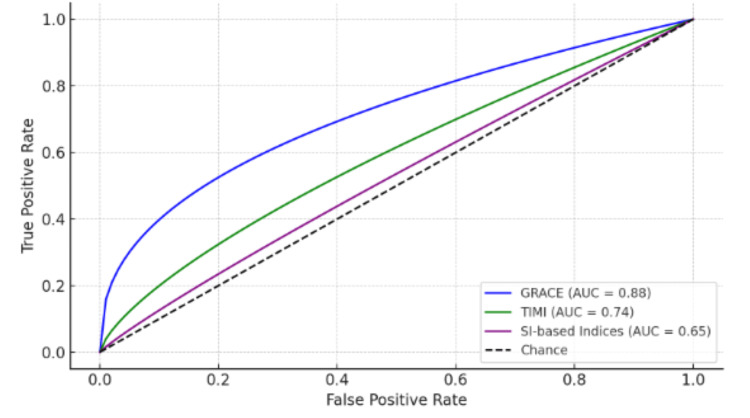
ROC curves for mortality and MACE prediction AUC: area under the curve; GRACE: Global Registry of Acute Coronary Events; MACE: major adverse cardiac events; ROC: receiver operating characteristic; SI: Shock Index; TIMI: Thrombolysis in Myocardial Infarction

**Figure 2:**
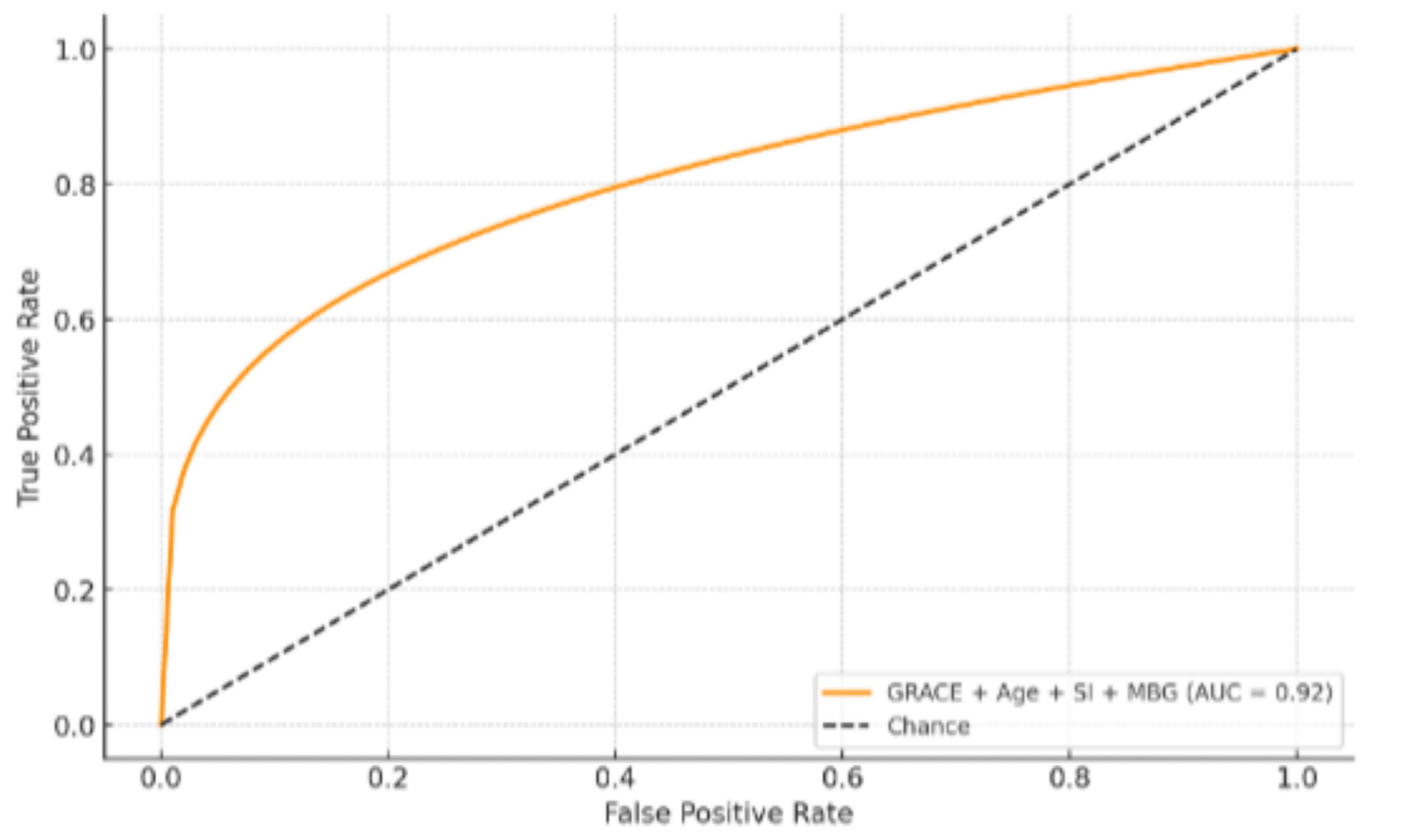
Combined model (GRACE + Age + SI + MBG) ROC curve AUC: area under the curve; GRACE: Global Registry of Acute Coronary Events; MBG: Myocardial Blush Grade; ROC: receiver operating characteristic; SI: Shock Index; TIMI: Thrombolysis in Myocardial Infarction

ROC curve analysis (Figure 1) demonstrated that GRACE possessed greater discriminatory ability for mortality (AUC = 0.88, 95% CI: 0.81-0.94) and MACE (AUC = 0.82, 95% CI: 0.75-0.89) than TIMI (mortality AUC = 0.74, p = 0.01; MACE AUC = 0.69, p = 0.03) and SI-based indices (mortality AUC = 0.65, p = 0.002). The addition of age, SI, and MBG to GRACE additionally enhanced mortality prediction (AUC = 0.92, 95% CI: 0.87-0.97) (Figure 2).

Multivariate Predictors

GRACE score (OR = 3.2, p<0.001), anterior STEMI (OR = 2.1, p = 0.04), and MBG ≤1 (OR = 2.8, p = 0.009) independently predicted mortality in logistic regression (Table 5).

**Table 5:** Multivariate logistic regression analysis for mortality Global Registry of Acute Coronary Events; MBG: Myocardial Blush Grade; STEMI: ST-segment elevation myocardial infarction

Predictor variable	Odds ratio (OR)	P-value
GRACE score	3.2	<0.001
Anterior STEMI	2.1	0.04
MBG ≤1	2.8	0.009

The result shows that the GRACE risk score possesses better prognostic value for mortality and 30-day MACE in STEMI patients who are being reperfused than TIMI and SI-based scores, with its high-risk stratification (>140) demonstrating the best correlation with unfavorable outcomes (p<0.001). Whereas SI and MBG offered limited independent predictive ability, their combination with GRACE substantially improved risk discrimination (AUC = 0.92), highlighting the clinical utility of merging dynamic hemodynamic and angiographic markers with traditional risk scores. These results endorse the priority application of GRACE in the acute environment and underscore the importance of prospective validation of hybrid models to refine risk prediction and inform individualized therapeutic approaches in STEMI management.

## Discussion

This study assessed the clinical profile, risk stratification, and outcome of 150 STEMI patients in a tertiary care center with special emphasis on TIMI and GRACE risk scores for adverse event prediction. The results are in accordance with past studies, and we provide additional information on risk assessment and the prognostic value of these scoring systems in STEMI patients.

Our research identified that 52% of patients were at intermediate TIMI risk [4-6], and 22% were high-risk according to GRACE (>140). These findings are in line with Yanqiao et al. (2022), who showed that GRACE scores are more likely to classify patients as high-risk than TIMI in East Asian populations, especially for long-term mortality prediction [17]. Similarly, Ji et al. (2023) reported that GRACE outperformed TIMI in predicting short-term mortality post-PCI, reinforcing its utility in acute settings [18]. Its greater sensitivity in detecting high-risk patients can be explained by the fact that it incorporates other variables like creatinine and systolic blood pressure, while TIMI relies more on the clinical presentation and comorbidities. This is consistent with Wu et al. (2019), who discovered that GRACE was better than TIMI in predicting in-hospital death in Chinese NSTEMI patients, reflecting its wider application among various acute coronary syndromes [19].

Our research recorded a 12% mortality and 16% 30-day MACE rate, which were largely caused by heart failure (6.7%) and reinfarction (5.3%). The results are in line with Hsieh et al. (2017), who reported that elevated GRACE scores were significantly associated with adverse long-term outcomes, even among patients without atrial fibrillation [20]. Our cohort's greater MACE rates could be attributed to the older patient population (26.7% >65 years) and the presence of significant comorbidities (DM: 41.3%, HTN: 52%), known predictors of adverse outcomes. The dominance of anterior STEMI (43.3%) and LAD involvement (48%) in our study is in line with international registries, such as the CAMI registry (Wu et al., 2019), which also documented similar trends among Chinese STEMI patients [19]. The increased frequency of sedentary lifestyle (69.3%) and smoking (56%) further highlights the role of modifiable risk factors in STEMI occurrence, emphasizing the importance of aggressive secondary prevention.

Our results validate the practice of using GRACE scores regularly for risk stratification in STEMI patients, especially in determining those with the greatest risk of mortality and MACE. The TIMI score, although helpful with initial risk prediction, may be augmented with GRACE or other scoring systems in high-risk individuals. The high incidence of heart failure after STEMI (6.7%) also underscores the need for early echocardiographic evaluation and guideline-mediated medical treatment.

Limitations

This single-site study design limits the generalizability of our findings. Additionally, the retrospective nature of data collection introduces the potential for selection bias. The relatively short duration of follow-up restricts our ability to assess long-term outcomes, particularly late MACE. Despite these limitations, our analysis demonstrates that in STEMI patients, the GRACE risk score showed superior prognostic performance compared to the TIMI score, particularly in identifying high-risk individuals. The high prevalence of modifiable risk factors such as smoking and physical inactivity underscores the critical need for robust secondary prevention strategies. Future research should explore integrated risk models that combine TIMI and GRACE scores with imaging data to enhance risk stratification and prediction accuracy.

## Conclusions

In this prospective observational study of STEMI patients receiving reperfusion therapy in a tertiary care center, GRACE scoring was found to have better prognostic performance than TIMI for predicting adverse outcomes, especially death and 30-day MACE. The superior predictive value of GRACE can be explained by its incorporation of extensive clinical and laboratory parameters. The observed significant prevalence of modifiable risk factors like sedentary lifestyle and smoking also reiterates the urgent requirement for preventive measures in this group. The results endorse regular use of GRACE scoring in acute care settings for successful risk stratification and emphasize the significance of implementing integrated strategies for improved clinical decision-making in STEMI care.

## References

[1] Handayani A, Kaban K, Nasri M, Mukhtar Z, Siregar AA (2017). Shock index as a simple clinical independent predictor of in-hospital major adverse cardiac events in non-ST-elevation myocardial infarction patients presenting with heart failure. Indonesian J Cardiol.

[2] Abreu G, Azevedo P, Galvão Braga C (2018). Modified shock index: a bedside clinical index for risk assessment of ST-segment elevation myocardial infarction at presentation. Rev Port Cardiol (Engl Ed).

[3] Kozieradzka A, Kamiński KA, Maciorkowska D (2011). GRACE, TIMI, Zwolle and CADILLAC risk scores--do they predict 5-year outcomes after ST-elevation myocardial infarction treated invasively?. Int J Cardiol.

[4] D'Ascenzo F, Biondi-Zoccai G, Moretti C (2012). TIMI, GRACE and alternative risk scores in acute coronary syndromes: a meta-analysis of 40 derivation studies on 216,552 patients and of 42 validation studies on 31,625 patients. Contemp Clin Trials.

[5] Lev EI, Kornowski R, Vaknin-Assa H (2008). Comparison of the predictive value of four different risk scores for outcomes of patients with ST-elevation acute myocardial infarction undergoing primary percutaneous coronary intervention. Am J Cardiol.

[6] Aragam KG, Tamhane UU, Kline-Rogers E (2009). Does simplicity compromise accuracy in ACS risk prediction? A retrospective analysis of the TIMI and GRACE risk scores. PLoS One.

[7] Morrow DA, Antman EM, Charlesworth A (2000). TIMI risk score for ST-elevation myocardial infarction: a convenient, bedside, clinical score for risk assessment at presentation: an Intravenous Tissue Plasminogen Activator for Treatment of Infarcting Myocardium Early II Trial Substudy. Circulation.

[8] Granger CB, Goldberg RJ, Dabbous O (2003). Predictors of hospital mortality in the Global Registry of Acute Coronary Events. Arch Intern Med.

[9] Addala S, Grines CL, Dixon SR (2004). Predicting mortality in patients with ST-elevation myocardial infarction treated with primary percutaneous coronary intervention (PAMI risk score). Am J Cardiol.

[10] Halkin A, Singh M, Nikolsky E (2005). Prediction of mortality after primary percutaneous coronary intervention for acute myocardial infarction: the CADILLAC risk score. J Am Coll Cardiol.

[11] Mutschler M, Nienaber U, Münzberg M (2013). The Shock Index revisited - a fast guide to transfusion requirement? A retrospective analysis on 21,853 patients derived from the TraumaRegister DGU. Crit Care.

[12] Berger T, Green J, Horeczko T (2013). Shock index and early recognition of sepsis in the emergency department: pilot study. West J Emerg Med.

[13] Yu T, Tian C, Song J, He D, Sun Z, Sun Z (2017). Age shock index is superior to shock index and modified shock index for predicting long-term prognosis in acute myocardial infarction. Shock.

[14] Zhou J, Shan PR, Xie QL, Zhou XD, Cai MX, Xu TC, Huang WJ (2019). Age shock index and age-modified shock index are strong predictors of outcomes in ST-segment elevation myocardial infarction patients undergoing emergency percutaneous coronary intervention. Coron Artery Dis.

[15] Shangguan Q, Xu JS, Su H, Li JX, Wang WY, Hong K, Cheng XS (2015). Modified shock index is a predictor for 7-day outcomes in patients with STEMI. Am J Emerg Med.

[16] van 't Hof AW, Liem A, Suryapranata H, Hoorntje JC, de Boer MJ, Zijlstra F (1998). Angiographic assessment of myocardial reperfusion in patients treated with primary angioplasty for acute myocardial infarction: myocardial blush grade. Zwolle Myocardial Infarction Study Group. Circulation.

[17] Yanqiao L, Shen L, Yutong M, Linghong S, Ben H (2022). Comparison of GRACE and TIMI risk scores in the prediction of in-hospital and long-term outcomes among East Asian non-ST-elevation myocardial infarction patients. BMC Cardiovasc Disord.

[18] Ji C, Song F, Huang X, Qu X, Qiu N, Zhu J (2023). Comparison of the predictive value of the modified CADILLAC, GRACE and TIMI risk scores for the risk of short-term death in patients with acute ST segment elevation myocardial infarction after percutaneous coronary intervention (Article in Chinese). Zhonghua Wei Zhong Bing Ji Jiu Yi Xue.

[19] Wu C, Gao XJ, Zhao YY (2019). Prognostic value of TIMI and GRACE risk scores for in-hospital mortality in Chinese patients with non-ST-segment elevation myocardial infarction (Article in Chinese). Zhonghua Xin Xue Guan Bing Za Zhi.

[20] Hsieh MJ, Lee CH, Chen CC, Chang SH, Wang CY, Hsieh IC (2017). Predictive performance of HAS-BLED risk score for long-term survival in patients with non-ST elevated myocardial infarction without atrial fibrillation. J Cardiol.

